# The Flower-Infecting Fungus *Ustilaginoidea virens* Subverts Plant Immunity by Secreting a Chitin-Binding Protein

**DOI:** 10.3389/fpls.2021.733245

**Published:** 2021-08-06

**Authors:** Guo-Bang Li, Jing Fan, Jin-Long Wu, Jia-Xue He, Jie Liu, Shuai Shen, Zeeshan Ghulam Nabi Gishkori, Xiao-Hong Hu, Yong Zhu, Shi-Xin Zhou, Yun-Peng Ji, Mei Pu, Jing-Hao Zhao, Zhi-Xue Zhao, He Wang, Ji-Wei Zhang, Yan-Yan Huang, Yan Li, Fu Huang, Wen-Ming Wang

**Affiliations:** State Key Laboratory of Crop Gene Exploration and Utilization in Southwest China, Sichuan Agricultural University, Chengdu, China

**Keywords:** chitin-triggered immunity, chitin receptor, flower-infecting, rice false smut, *Ustilaginoidea virens*

## Abstract

*Ustilaginoidea virens* is a biotrophic fungal pathogen specifically colonizing rice floral organ and causes false smut disease of rice. This disease has emerged as a serious problem that hinders the application of high-yield rice cultivars, by reducing grain yield and quality as well as introducing mycotoxins. However, the pathogenic mechanisms of *U. virens* are still enigmatic. Here we demonstrate that *U. virens* employs a secreted protein UvCBP1 to manipulate plant immunity. *In planta* expression of *UvCBP1* led to compromised chitin-induced defense responses in *Arabidopsis* and rice, including burst of reactive oxygen species (ROS), callose deposition, and expression of defense-related genes. *In vitro*-purified UvCBP1 protein competes with rice chitin receptor OsCEBiP to bind to free chitin, thus impairing chitin-triggered rice immunity. Moreover, *UvCBP1* could significantly promote infection of *U. virens* in rice flowers. Our results uncover a mechanism of a floral fungus suppressing plant immunity and pinpoint a universal role of chitin-battlefield during plant–fungi interactions.

## Introduction

As a staple food, rice feeds over half of the population of the world. Rice diseases have caused significant yield losses every year, thus being urgent problems to ensure food security (Liu et al., 2014). Rice false smut, caused by the fungus *Ustilaginoidea virens* (teleomorph: *Villosiclava virens*), emerges as one of the most devastating diseases for rice production (Sun et al., 2020). *U. virens* specifically infects rice flowers and develops into large mycelial colonies covered with massive chlamydospores, called false smut balls (Tang et al., 2013; Fan et al., 2016). Formation of false smut balls not only leads to considerable yield losses but also generates a diversity of mycotoxins poisonous to humans and animals (Zhou et al., 2012; Dhua et al., 2015). *U. virens*-produced mycotoxins mainly include ustiloxins and ustilaginoidins, which show cytotoxicities and phytotoxicities by inhibiting the mitosis process in eukaryotic cells (Zhou et al., 2012). Despite the notorious effects, little is known on the pathogenesis and resistance mechanisms of rice false smut. Resistance genes against *U. virens* have not been cloned in rice or its relatives, except that a candidate gene (LOC_Os01g42630) was suggested (Wang et al., 2019; Qiu et al., 2020). Development of disease intervention strategies will benefit from understanding the pathogenic mechanisms of *U. virens*.

*Ustilaginoidea virens* infects rice flowers specifically at the late booting stage of rice (Sun et al., 2020). At around 1 week before heading, *U. virens* spores initiate infection by germinating on the surface of developing spikelets, and then the hyphae grow epiphytically without penetrating the spikelet tissues and cells (Fan et al., 2014, 2015). Extending hyphae reach to the inner space of a spikelet through the gap between the lemma and the palea (Ashizawa et al., 2012), and preferentially attack stamen filaments intercellularly (Tang et al., 2013). This infection stage seems to be vital for successful colonization of *U. virens*, as stamen-defect rice mutant could not support disease progression of false smut (Fan et al., 2020). After colonization in stamen filaments, *U. virens* hyphae extend to other floral tissues, including anthers, lodicules, stigmas, and ovary, and only infect lodicules and stigmas to a lesser extent (Tang et al., 2013; Song et al., 2016). Finally, *U. virens* mycelia grow large to a false smut ball, probably via hijacking rice grain-filling system to obtain abundant nutrients (Fan et al., 2015; Song et al., 2016).

The specific infection strategy of *U. virens*, as revealed by above cellular studies, drives researchers to explore the underlying molecular mechanisms between *U. virens* and rice. Decoding of *U. virens* genome reveals that *U. virens* possesses no pectin lyases and reduced gene inventory for glycoside hydrolases, which are involved in breaking down of plant cell wall components (Zhang et al., 2014). This finding is in accordance with the intercellular infection mode in stamen filaments, which are arranged loosely (Rong et al., 2017), suggesting adaptations of *U. virens* to flower infection and biotrophic lifestyle. As a biotroph, *U. virens* must be able to escape or subvert the surveillance of host immune system. Pathogen effectors play a vanguard role in manipulating plant immunity for promoting colonization (Toruño et al., 2016; Wang and Wang, 2018). The effector pool in *U. virens* contains 256 putative effector proteins with signal peptides (SPs) and 165 unconventional effectors with rich Cys residues (Zhang et al., 2021). A small set of effectors have been demonstrated to suppress cell death in *Nicotiana benthamiana* triggered by *Burkholderia glumae* (Zhang et al., 2014). Among them, SCRE1 and SCRE2 also suppress Bax- and INF1-induced cell death and attenuate pathogen-associated molecular pattern (PAMP)-triggered immunity in rice (Fang et al., 2019; Zhang et al., 2020). In addition, the core effector *SCRE2*/*UV_1261* contributes to *U. virens* pathogenicity in rice panicles (Fan et al., 2019; Fang et al., 2019). However, it remains elusive how *U. virens* effectors counteract plant immunity.

To understand the pathogenesis mechanisms of rice false smut, we initially performed a genome-wide transcriptional analysis on rice flowers infected with *U. virens*, and found that *U. virens* infection suppressed the expression of defense-related genes, such as *NPR1* and *CNGC*, in rice flowers (Fan et al., 2015). In this study, we demonstrated that *U. virens* highly expressed a secreted protein during infection, which could bind with free chitin to sequester chitin-triggered plant immunity and promote *U. virens* colonization. Our findings provide new insights into the pathogenic mechanisms of a floral organ-specific pathogen.

## Materials and Methods

### Plant Materials and Fungal Strains

In this study, rice cultivar Q455 was used for genetic transformation and grown in an experimental field under natural conditions. Col-gl (*Arabidopsis thaliana* accession Col-0 containing the glabrous mutation) was used for genetic transformation and grown in a growth chamber under conditions of short day (8 h of light and 16 h of darkness), 22°C, 75% relative humidity (RH). *N. benthamiana* plants were grown at 22°C, 75% RH under 10 h light and 14 h darkness. *U. virens* isolate PJ52-2-5 (PJ52 for short) was used for artificial inoculation experiments. PJ52 was cultured at 28°C in potato sucrose agar (PSA: 200 g/L potato, 20 g/L sucrose, 13 g/L agar) or potato sucrose (PS: 200 g/L potato, 20 g/L sucrose) media. *Rhizoctonia solani* isolate AG1-IA was cultured at 28°C in potato dextrose agar (PDA: 200 g/L potato, 20 g/L dextrose, 13 g/L agar) medium.

### Constructs and Transformation

For validation of UvCBP1 SP, predicted UvCBP1^*SP*^ sequence was synthesized by Sangon Biotech (Chengdu, China) and cloned into the pSUC2T7M13ORI (pSUC2) vector (Jacobs et al., 1997) to generate pSUC2-UvCBP1^*SP*^.

For purification of recombinant proteins, sequences encoding mature UvCBP1 and OsCEBiP proteins were amplified with indicated primers (Supplementary Table 1) and ligated into the *Bam*HI-*Eco*RI linearized pMAL-c5x or pGEX-6p-1 vectors.

For transient expression in *N. benthamiana* and stable expression in *A. thaliana*, the full-length of *UvCBP1* was amplified from PJ52 with the primer pair UvfCBP1_SacIF/UvfCBP1_KpnIR (Supplementary Table 1). The resultant was ligated into the binary vector pCAMBIA1300-eYFP to generate *UvCBP1-eYFP* construct, which was introduced to *Agrobacterium tumefaciens* strain GV3101. Transformation in *N. benthamiana* or *Arabidopsis* was performed as described (Clough and Bent, 1998; Huang et al., 2014).

For stable expression in rice, full-length of *UvCBP1* was amplified with the primer pair UvfCBP1_SacIF/UvfCBP1_SpelIR (Supplementary Table 1) and ligated to pCAMBIA1300 to generate *35S-UvCBP1* construct. EHA105 containing *35S-UvCBP1* was introduced into rice cultivar Q455. Positive transgenic plants were screened by hygromycin resistance analysis (Li et al., 2014).

### Fungal Inoculation

*Ustilaginoidea virens* inoculation was conducted as previously described (Fan et al., 2015). Briefly, rice panicles at late booting stage (approximately 1 week before heading) were inoculated with blended mixture of *U. virens* mycelia and conidia, whose concentration was adjusted to 1 × 10^6^ conidia/mL. Inoculated rice panicles were sampled at 1, 3, 5, 7, 9, 11, 13, and 15 dpi (days post inoculation) for reverse transcription-quantitative polymerase chain reaction (RT-qPCR). At around 24 dpi, diseased panicles were photographed and the number of false smut balls of each panicle was recorded. *R. solani* inoculation was followed by the method described by Zheng et al. (2013).

### Verification of Signal Peptide

To verify the functionality of UvCBP1 SP, plasmids of pSUC2-UvCBP1^*SP*^, pSUC2-Avr1b^*SP*^, and pSUC2-Mg87^*N*^ were transformed into yeast strain YTK12 and subjected to yeast secretion assay as performed previously (Fan et al., 2019). To further confirm whether UvCBP1 could be secreted, subcellular localization of UvCBP1-eYFP was determined in *N. benthamiana*. After transient expression of UvCBP1-eYFP or eYFP control in *N. benthamiana* for 2 days, leaf discs were treated with 10% (m/v) sucrose solution for 10 min to achieve plasmolysis and then viewed under a laser scanning confocal microscope (Nikon A1). Protein size was confirmed by a western blot assay with an anti-GFP antibody (BBI Life Science).

### RNA Extraction and RT-qPCR

*Arabidopsis* leaves were infiltrated with 10 μg/mL of chitin, and samples were collected at 12 h post infiltration for subsequent experiments. Rice leaves or spikelets were incubated with 30 μg/mL of chitin alone or chitin preincubated with 30 μg recombinant protein (Glutathione S-Transferase (GST) or GST-UvCBP1), and sampled at different time points. Total RNA was extracted using the TRIzol Reagent (Invitrogen) and the first-strand cDNA was synthesized using the ReverTra Ace kit (TOYOBO). SYBR Green mix (Takara) was used with corresponding primers (Supplementary Table 1) for expression analysis. *AtACT2*, *OsUbi*, and *UvTub2*α were used as internal controls for quantifying gene expression in *Arabidopsis*, rice, and *U. virens*, respectively.

### Measurement of Reactive Oxygen Species

In *Arabidopsis*, chitin-induced burst of reactive oxygen species (ROS) was measured as described by Li et al. (2018). In rice, chitin was incubated with the recombinant protein (GST-UvCBP1 or GST) for 1 h, and then mixed with 20 μM luminol and 10 μg/mL horseradish peroxidase. The resultants were applied to rice leaf discs to induce ROS, which was recorded in a GloMax 20/20 luminometer (Shi et al., 2018).

### Callose Deposition

In *Arabidopsis*, chitin-induced callose deposition was performed as described by Li G.-B. et al. (2020). In rice, the first healthy leaves of 5-day-old seedling were collected and placed in ddH_2_O for 6 h. Chitin solution (final concentration 50 μg/mL) was added and subjected to vacuum treatment for 40 min, prior to 20 h incubation at room temperature. Subsequently, aniline blue staining for callose deposits was conducted according to a previous work (Liu et al., 2012).

### Chitin Binding Assay

The recombinant proteins (GST-UvCBP1 and GST) were purified from *Escherichia coli* and used for chitin-binding assay as described earlier with modifications (Han et al., 2019). Briefly, the recombinant proteins were incubated with chitin beads (NEB), crab shell chitin (Sigma), cellulose (Sigma), and chitosan (Sigma) in 800 μL ddH_2_O at 4°C. After 4 h, the insoluble pellet fraction was centrifuged (4°C, 12000 rpm, 10 min), and the supernatant was collected. The insoluble pellets were rinsed three times with ddH_2_O. Both the supernatants and the pellets were boiled in 1% sodium dodecyl sulfate (SDS) for extraction of proteins, which were then separated in 10% SDS-polyacrylamide gel electrophoresis (PAGE) gels and immunoblotted with an anti-GST antibody (Invitrogen).

### Phylogenetic Analysis

Genes encoding proteins MoChia1 from *Magnaporthe oryzae*, MrChi from *Moniliophthora roreri*, MpChi from *Moniliophthora perniciosa*, and other paralogs of *UvCBP1* from *U. virens* were retrieved from the UniProt database website^1^. The amino acid (aa) sequences were aligned by the MEGA5.1 software (Tamura et al., 2011). The resultant file was subjected to iTOL (Letunic and Bork, 2016)^2^ for construction of phylogenetic tree with default parameters.

## Results

### *UvCBP1* Encodes a Secreted Protein and Is Highly Expressed During *Ustilaginoidea virens* Infection

**FIGURE 1:**
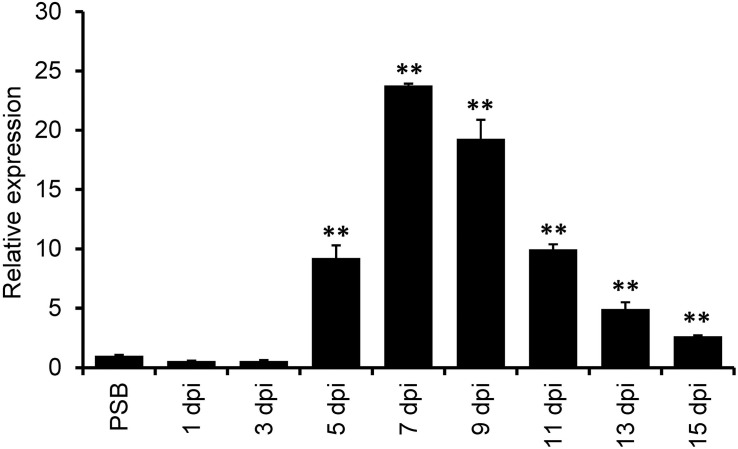
Expression analysis of *UvCBP1* during *Ustilaginoidea virens* infection in rice. Spikelets from PJ52-inoculated rice panicles were sampled at indicated time points and subjected to RT-qPCR analysis. The mixture of mycelia and conidia from PSB-cultured PJ52 was collected as the control sample. Relative expression level of *UvCBP1* was determined using *UvTub2*α as the reference gene. Data are represented as means ± SD of three biological replicates. Asterisk indicates significant difference determined by Student’s *t* test (^∗∗^*P* < 0.01). Similar results were obtained from two independent experiments. dpi, day post inoculation.

A previous *de novo* transcriptome analysis identified a number of *U. virens* genes highly expressed during infection with rice flowers (Fan et al., 2015). Here, we selected one of the top 10-highly induced gene *UvCBP1* for further investigation. We first determined a time-course expression pattern of *UvCBP1* by RT-qPCR analysis. Compared to the expression level of *UvCBP1* in axenic culture, the abundance of *UvCBP1* transcripts started to increase at 5 dpi, peaked at 7 dpi with more than 20-fold increase when *U. virens* hyphae invaded the inner floral organs of rice spikelets (Figure 1) (Fan et al., 2015).

**FIGURE 2:**
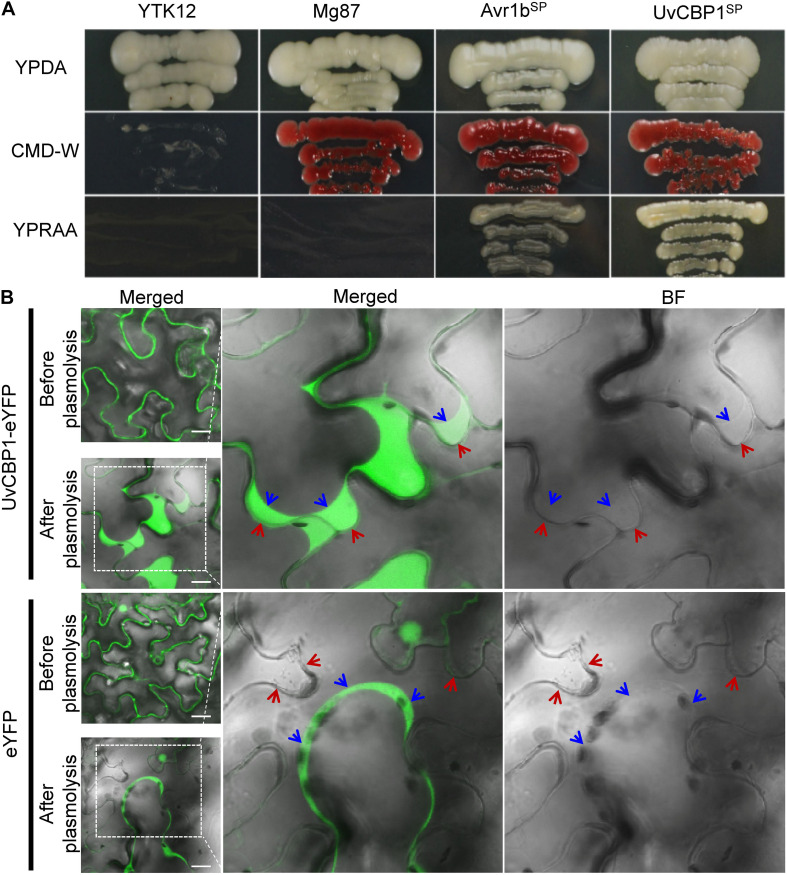
*UvCBP1* encodes a secreted protein. **(A)** Validation of UvCBP1 signal peptide (SP). The DNA fragment encoding SP of UvCBP1 was cloned into pSUC2, in frame with an invertase gene. The resultant plasmid was transformed into YTK12 that is unable to utilize raffinose. SP of UvCBP1 could enable YTK12 to grow on YPRAA medium, indicating its functionality. SP of Avr1b and N-terminus of Mg87 were applied as positive and negative controls, respectively. **(B)** UvCBP1-eYFP is located in the apoplast of leaf cells in *Nicotiana benthamiana.* UvCBP1-eYFP or eYFP was transiently expressed in *N. benthamiana* and examined by confocal microscopy. After plasmolysis with 10% sucrose, fluorescence signal of UvCBP1-eYFP was clearly detected in the extracellular space, while eYFP was in the cytoplasm and nucleus. The blue arrows indicate plasma membrane and the red arrows indicate plant cell wall. Scale bar, 20 μm.

Sequence analysis revealed that *UvCBP1* encoded a putative protein with 450 aa containing a predicted SP (Supplementary Figure 1). The functionality of UvCBP1-SP was verified by a yeast secretion assay as described previously (Jacobs et al., 1997; Fang et al., 2016; Fan et al., 2019). The sequence encoding predicted SP of UvCBP1 was fused in frame with mature invertase (SUC2) and introduced into the yeast strain YTK12. The wild-type YTK12 cannot utilize raffinose due to its deficiency in invertase secretion, whereas the YTK12 strain transformed with the *UvCBP1^*SP*^-SUC2* could grow well on YPRAA medium supplemented with raffinose as the sole carbon source. YTK12 strains transformed with *Avr1b^*SP*^-SUC2* or *Mg87 ^*N–terminus*^-SUC2* were used as positive and negative control, respectively (Figure 2A). As a result, UvCBP1^*SP*^ is a functional SP.

To further verify UvCBP1 as a secreted protein, we tagged the full-length of UvCBP1 with a yellow fluorescent protein (UvCBP1-eYFP) and expressed it in *N. benthamiana*. After confirmation of expression of indicated proteins by western blot (Supplementary Figure 2A), we examined the subcellular localization of expressed proteins. Assisted by plasmolysis treatment with 10% sucrose, we observed that UvCBP1-eYFP was located in the apoplast space between *N. benthamiana* cells. As a control, eYFP was located in the cytoplasm and the nucleus of plant cells (Figure 2B). Taken together, *UvCBP1* encodes a secreted protein that is likely involved in *U. virens* infection.

### *In planta* Expression of *UvCBP1* Suppresses Chitin-Triggered Plant Immunity

**FIGURE 3:**
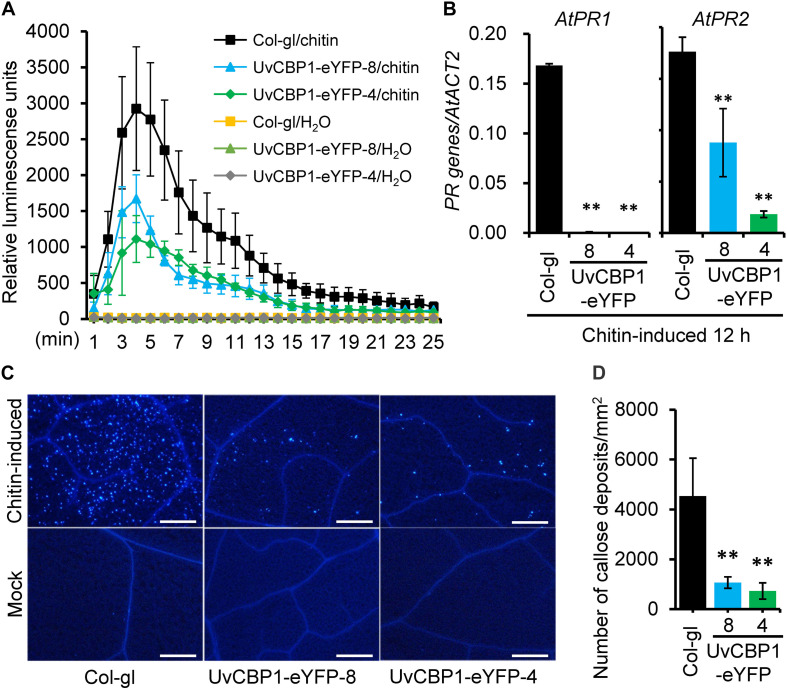
*UvCBP1* suppresses chitin-triggered immunity in *Arabidopsis thaliana.* **(A)** Chitin-induced burst of ROS. Data were represented as the mean ± SD of four biological replicates. **(B)** Expression analysis of defense-related genes in chitin-treated leaves of *UvCBP1*-expressing plants and Col-gl control. Relative expression levels of indicated genes were determined using *AtACT2* as the reference gene. Data were represented as the mean ± SD of three replicates. **(C,D)** Chitin-induced callose deposition. Leaves of indicated *Arabidopsis* plants were infiltrated with chitin. At 10 h post infiltration, leaves were collected for aniline blue staining. Representative microscopy images were shown **(C)** and the number of callose deposits was recorded **(D)**. Bar data were mean ± SD of four biological replicates. Scale bar, 50 μm. Asterisk in this figure indicates significant difference determined by Student’s *t* test (^∗∗^*P* < 0.01).

Highly induced expression of *UvCBP1 in planta* suggests its role in communicating with plant host for the benefits of *U. virens*. To explore the functions of *UvCBP1 in planta*, we first generated *A. thaliana* transgenic plants expressing the full-length of *UvCBP1-eYFP*. Positive transgenic plants were confirmed by western blot with an anti-GFP antibody (Supplementary Figure 2B). We then analyzed the effects of *UvCBP1-eYFP* on chitin-triggered immunity in *Arabidopsis*. In the wild-type Col-gl, chitin induced rapid production of ROS, high expression of defense-related genes *AtPR1* and *AtPR2*, and massive accumulation of callose deposits in leaves (Figure 3). By contrast, expression of *UvCBP1-eYFP* significantly inhibited chitin-induced ROS burst, *PR* gene expression, and callose deposition, compared with those in Col-gl controls (Figure 3).

**FIGURE 4:**
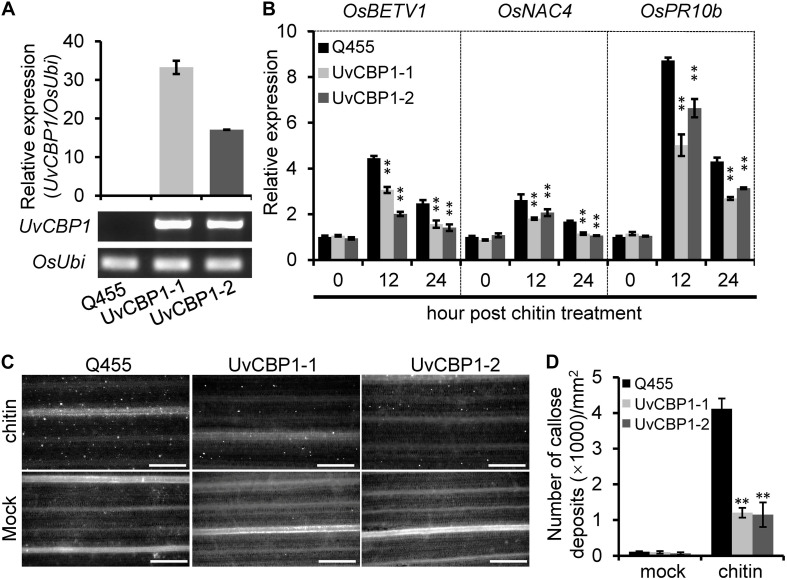
*In planta* expression of *UvCBP1* compromises rice immunity triggered by chitin. **(A)** Expression analysis of *UvCBP1* in transgenic rice plants. The relative expression was determined by RT-qPCR using *OsUbi* as the reference gene. No expression of *UvCBP1* was detected in the wild-type Q455. **(B)** Expression analysis of defense-related genes in chitin-treated leaves of *UvCBP1*-expressing plants and Q455. Relative expression levels of indicated genes were determined using *OsUbi* as the reference gene. Data are represented as means ± SD of three biological replicates. **(C,D)** Chitin-induced callose deposition. Leaves of indicated rice plants were treated with chitin for 20 h and subjected to aniline blue staining. Representative microscopy images were shown **(C)** and the number of callose deposits was recorded **(D)**. Bar data were mean ± SD of four biological replicates. Scale bar, 50 μm. Asterisk in this figure indicates significant difference determined by Student’s *t* test (^∗∗^*P* < 0.01).

These results drove us to further test whether *UvCBP1* could suppress chitin-triggered immunity in rice. We introduced the full-length of *UvCBP1* into the rice cultivar Q455, which is highly compatible with *U. virens*. We selected two independent transgenic lines with high expression of *UvCBP1* for subsequent analyses (Figure 4A and Supplementary Figure 3). In both leaf and floral organs, chitin induced the expression of defense-related genes, such as *OsBETV1*, *OsNAC4*, and *OsPR10b*. However, the induction of these genes was markedly suppressed in *UvCBP1*-expressing plants (Figure 4B and Supplementary Figure 3). Expression of *UvCBP1* in rice also inhibited chitin-triggered callose deposition (Figures 4C,D). These data support an important role of *UvCBP1* in subverting plant immunity.

### UvCBP1 Binds to Chitin and Attenuates Chitin Perception to Suppress Plant Immunity

**FIGURE 5:**
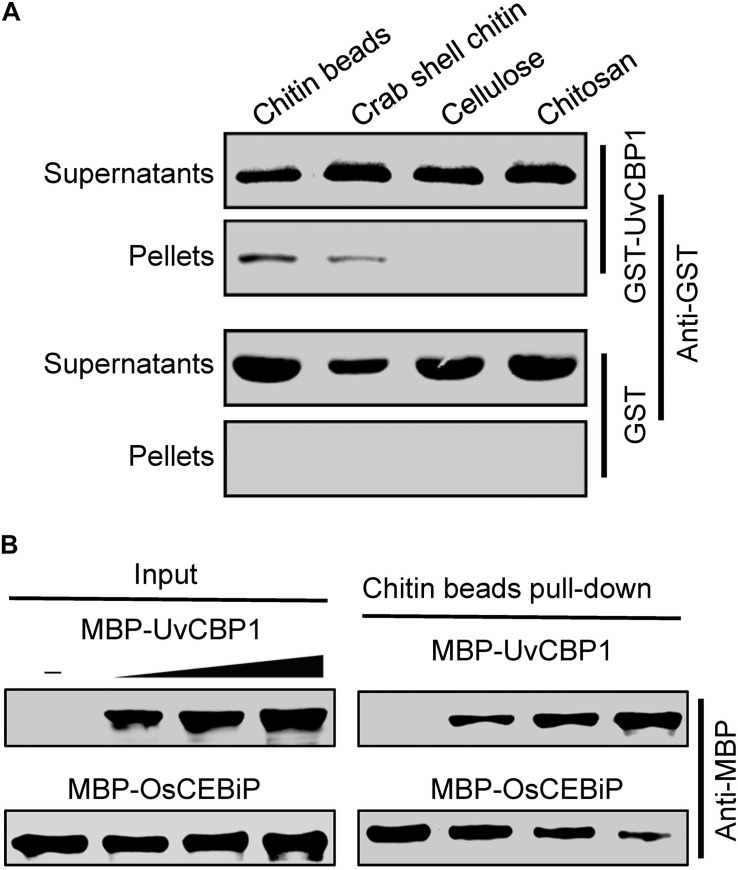
UvCBP1 competes with rice chitin receptor OsCEBiP to bind with free chitin. **(A)** Chitin-binding assay. The recombinant protein GST-UvCBP1 was incubated with insoluble chitin beads, shrimp shell, cellulose, and chitosan. GST protein was used as control. After incubation and elution, the pellets (P) and supernatant (S) were detected by Western blot with an anti-GST antibody. **(B)** UvCBP1 competes with OsCEBiP for chitin binding. Increasing amounts of MBP-UvCBP1 was mixed with MBP-OsCEBiP, and the resultants were incubated with chitin beads. MBP-UvCBP1 or MBP-OsCEBiP pull-down by chitin beads was detected with an anti-MBP antibody.

Homologous sequence search identified that UvCBP1 protein is homologous to several recently identified effectors, such as *M. oryzae* MoChia1, *M. perniciosa* MpChi, and *M. roreri* MrChi (Fiorin et al., 2018; Han et al., 2019). These effectors possess chitin-binding ability and thus could sequester chitin-triggered plant immunity. Nine aa residues were predicted to be chitin-binding residues in MpChi (Fiorin et al., 2018), and six of the nine residues were conserved in UvCBP1 (Supplementary Figure 1). It is indicated that UvCBP1 may be able to bind with chitin. To confirm this, we conducted a chitin-binding assay with GST-tagged UvCBP1 recombinant protein. After incubated with insoluble chitin beads, crab shell chitin, chitosan, or cellulose, GST-UvCBP1 could be coprecipitated with chitin beads and crab shell chitin, but not with chitosan and cellulose (Figure 5A). By contrast, the control GST protein was only detected in the supernatant and could not be coprecipitated with any of the tested cell wall polysaccharides (Figure 5A). As a result, UvCBP1 has chitin-binding activity. We assumed that UvCBP1 may compete with the rice chitin receptor OsCEBiP for chitin binding. To test this hypothesis, we incubated chitin beads with the recombinant protein MBP-OsCEBiP and increasing amounts of MBP-UvCBP1. After pull-down of the chitin beads, we detected decreasing amounts of OsCEBiP, correlated with increasing amounts of UvCBP1 (Figure 5B). This finding indicates that UvCBP1 could interfere with chitin perception by plant receptors.

**FIGURE 6:**
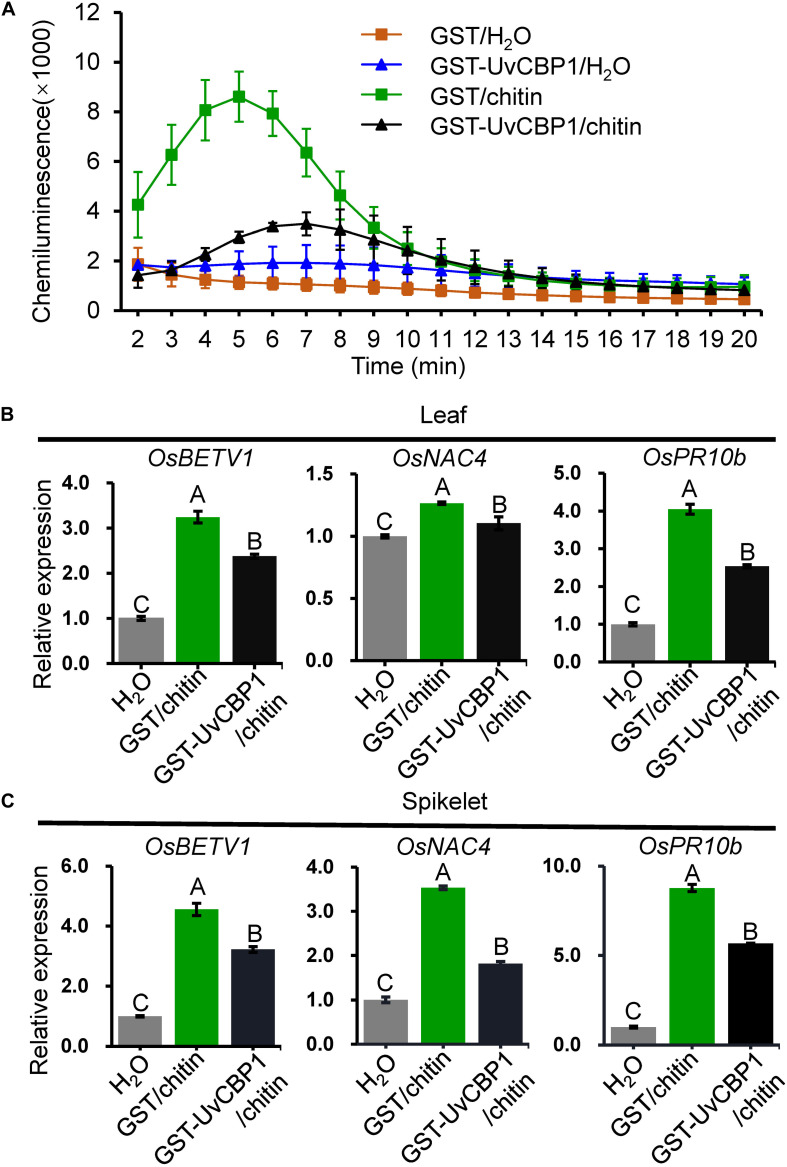
UvCBP1 traps chitin from activating defense responses in rice. **(A)** Chitin-induced ROS burst in rice leaves. Chitin was co-incubated with GST-UvCBP1 or GST for 1 h, and then the resultants were used for inducing ROS. The data indicate the mean ± SD of three biological replicates. The experiment was repeated twice with similar results. **(B,C)** Chitin-induced expression of defense-related genes in rice leaves **(B)** or spikelets **(C)**. Chitin was incubated with GST-UvCBP1 or GST for 1 h, and then the resultants were used to treat rice leaves (for 12 h) or spikelets (for 6 h). Relative expression of indicated genes was determined using *OsUbi* as the reference gene. Data are represented as mean ± SD of three replicates. Different letters above data bars indicate significant difference as determined by one-way ANOVA with *post hoc* Tukey honestly significant difference (HSD) analysis (*P* < 0.01).

Next, we tested whether *in vitro*-expressed GST-UvCBP1 could sequester chitin-induced defense responses in rice. Prior to treating rice, chitin was either incubated with GST-UvCBP1 or GST for 1 h. The results showed that rice leaves treated with GST-incubated chitin rapidly produced a great amount of ROS, whereas ROS burst occurred later and the amount was markedly reduced when chitin was incubated with GST-UvCBP1 (Figure 6A). Also, chitin-induced expression of defense genes, including *OsBETV1*, *OsNAC4*, and *OsPR10b*, was significantly suppressed by GST-UvCBP1 treatment (Figures 6B,C). These data indicate that UvCBP1 may impair chitin-OsCEBiP perception to attenuate chitin-induced immune responses in rice.

### *UvCBP1* Enhances Pathogen Infection in Rice

**FIGURE 7:**
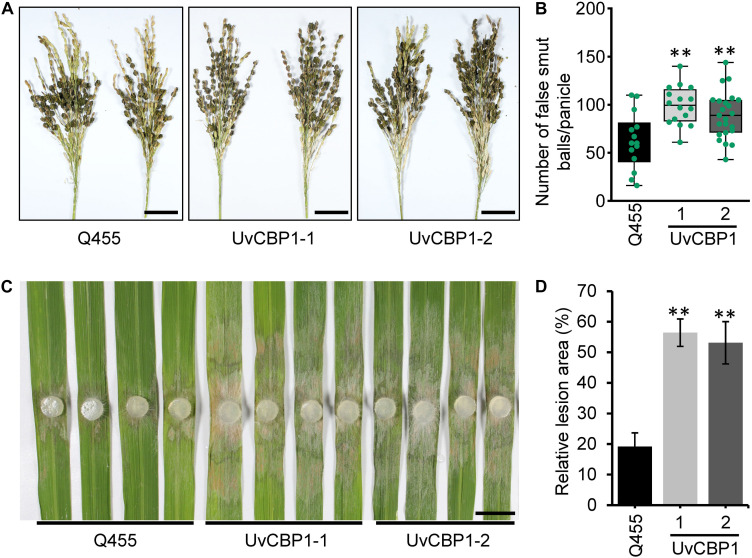
UvCBP1 enhances pathogen infection in rice. **(A,B)** Rice false smut disease assay. *U. virens* PJ52 was inoculated into rice panicles at late booting stage. Disease phenotype was recorded at 24 days post inoculation (dpi) **(A)**, and the number of false smut balls per panicle was box plotted **(B)**. Scale bar, 4 cm. **(C,D)** Rice sheath blight disease assay. Rice leaves were inoculated with mycelial plugs of *Rhizoctonia solani* strain AG1-IA. Disease phenotype was recorded at 2 dpi **(C)**, and the relative lesion area was calculated **(D)**. Scale bar, 2 cm. Asterisk in this figure indicates significant difference determined by Student’s *t* test (**P < 0.01).

To define the contribution of *UvCBP1* to pathogen colonization, we investigated whether *UvCBP1*-expressing rice lines are more sensitive to *U. virens*. At late booting stage of rice, the *U. virens* strain PJ52 were inoculated into rice panicles. After 24 days of inoculation, both *UvCBP1*-expressing rice and wild-type Q455 displayed false smut balls in panicles (Figure 7A), while *UvCBP1*-expressing rice panicles accommodated a larger number of false smut balls than Q455 panicles (Figure 7B), indicating that *UvCBP1* could promote *U. virens* infection in rice. As chitin-triggered immunity is a conserved antifungal pathway, we considered that *in planta* expression of *UvCBP1* may be able to compromise plant resistance to other fungal pathogens. In consistent with this notion, *UvCBP1*-expressing rice was more susceptible to a necrotrophic fungal pathogen, *R. solani*, which causes rice sheath blight disease (Figures 7C,D).

### *UvCBP1* Has Multiple Paralogs in *Ustilaginoidea virens*

**FIGURE 8:**
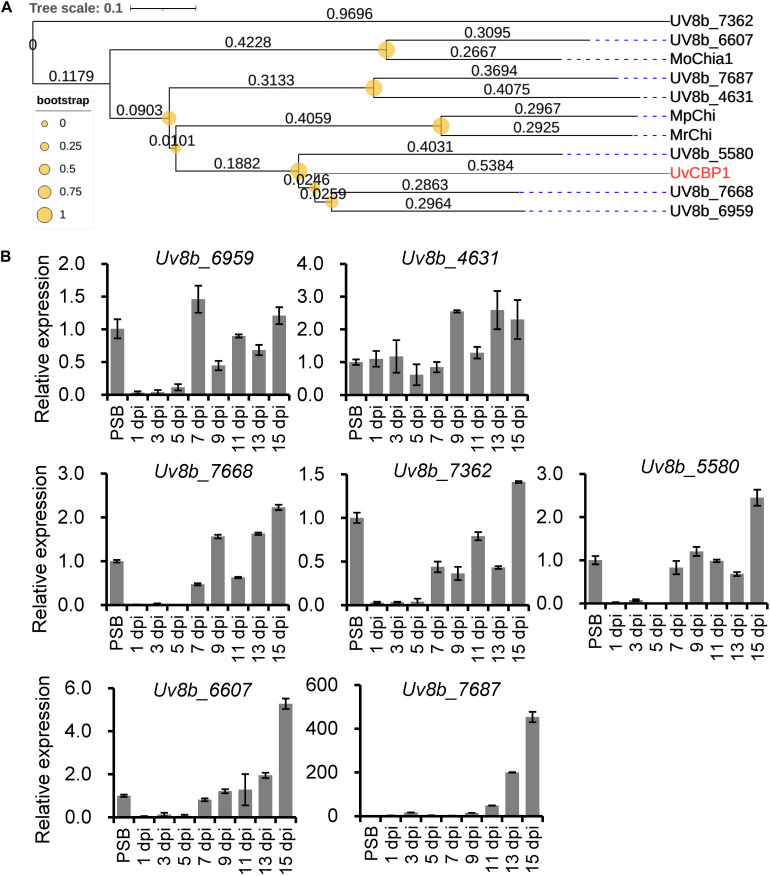
Phylogenetic and expression analyses of UvCBP1 paralogs in *U. virens*. **(A)** Phylogenetic analysis of UvCBP1 paralogs in *U. virens* and other fungal pathogens. The size of yellow circles represent bootstrap values ranging from 0 to 1. The numbers represent branch lengths. **(B)** Expression analysis of paralogous genes of *UvCBP1* during *U. virens* infection in rice. The mixture of mycelia and conidia from PS-cultured PJ52 was collected as the control sample. Relative expression levels of indicated genes were determined using *UvTub2*α as the reference gene. Data are represented as means ± SD of three biological replicates. dpi, days post inoculation.

Since *U. virens* infection process in rice flowers last for several weeks (Fan et al., 2020) and the expression of *UvCBP1* peaked at 7 dpi (Figure 1), it is possible that *U. virens* may deploy multiple effectors to synergically manipulate chitin-triggered rice immunity during the entire infection process. Indeed, we identified seven paralogs of *UvCBP1* in *U. virens* genome. Three of them were closely clustered with *UvCBP1* (Figure 8A), indicating that they may share similar biochemical functions. We next examined the expression patterns of *UvCBP1* paralogs during *U. virens* infection in rice flowers. Compared with the gene expression in PS culture, *Uv8b_6959* was slightly upregulated at 7 dpi, while other six paralogs tended to be induced at late stages of infection. Particularly, *Uv8b_7687* was induced by over 400-fold at 15 dpi (Figure 8B). Therefore, *UvCBP1* paralogs may contribute differently to *U. virens* colonization, and *UvCBP1* is likely the critical one contributing to the initial colonization in rice stamen filaments (Figure 1).

## Discussion

UvCBP1 is a secreted protein that is highly expressed particularly in the initial colonization stage in rice stamen filaments (Figure 1) (Fan et al., 2020). Although future work to knockout or knockdown *UvCBP1* in *U. virens* may reveal whether it is required for pathogenicity, we demonstrate that its *in planta* expression in rice spikelets enhances *U. virens* colonization (Figures 7A,B), supporting its role as a potential virulence factor. Furthermore, both *in planta* and *in vitro* data suggest that UvCBP1 could suppress plant pattern-triggered immunity, including ROS burst, callose deposition, and defense gene expression (Figures 3, 4, 6). Due to the chitin-binding ability, UvCBP1 acts as an effector protein to manipulate plant immunity.

Chitin-triggered immunity is a conserved antifungal immune system in plants, which may be subverted by sophisticated fungal pathogens via multiple strategies as summarized in a recent review (Gong et al., 2020). For instance, fungal pathogens protect their cell wall from releasing plant immunity-inducible chitin fragments by secreting proteases to degrade plant chitinases (Okmen et al., 2018), masking cell wall with α-1,3-glucan and effector proteins (van den Burg et al., 2006; Fujikawa et al., 2012), or deacetylating chitin to chitosan (Gao et al., 2019). Fungal pathogens can also secrete effectors with high affinity to chitin fragments, such as Ecp6 (de Jonge et al., 2010), Slp1 (Mentlak et al., 2012), MoAa91 (Li Y. et al., 2020), MpChi (Fiorin et al., 2018), MoChia1 (Han et al., 2019; Yang et al., 2019), and Mg3LysM (Marshall et al., 2011), to outcompete host chitin receptors and block chitin perception. Some powdery mildew fungi may deploy effectors with chitinase activity to degrade immunogenic chitin oligomers, and thus preventing perception by plant chitin receptors (Martínez-Cruz et al., 2021). Additionally, some fungi utilize effectors like NIS1 and AvrPiz-t to target plant intracellular immune components involved in chitin signaling, such as BIK1 and OsRac1 (Bai et al., 2019; Irieda et al., 2019). Although a set of effectors with diverse counteracting strategies have been identified, these findings are mostly restricted to foliar pathosystems. In this work, we show that the flower-infecting fungus *U. virens* sequesters chitin-triggered immunity by secreting UvCBP1 to bind with free chitin. Our findings support the idea that escaping or subverting chitin-induced plant immunity is a prerequisite for colonization of fungal pathogens in different plant organs.

The infection process of *U. virens* is generally consisted of two phases, including epiphytic growth stage on the surface of rice lemma and palea and intercellular infection stage in rice stamen and pistil organs (Song et al., 2016; Fan et al., 2020). Under our experimental conditions, the epiphytic stage usually lasts for approximately 5 days. Afterward, *U. virens* starts to colonize stamen filaments as primary infection sites. At 7 dpi, considerable infection hyphae can be detected at stamen filaments, indicating this time point may be critical for successful colonization of *U. virens* (Fan et al., 2020). In the present work, we detected an induced expression of *UvCBP1* as early as 5 dpi and the highest expression at 7 dpi (Figure 1). Due to the coincidence of *UvCBP1* expression and *U. virens* infection stage in stamen filaments, it is conceivable that UvCBP1 is a critical effector to manipulate rice immunity during initial intercellular infection of *U. virens*. By contrast, *UvCBP1* paralogs shared distinctive expression patterns from *UvCBP1* (Figure 8B). For example, *Uv8b_6607* and *Uv8b_7687* were mostly induced at a very late infection stage (15 dpi), when *U. virens* mycelia have developed into an early false smut ball (Wang et al., 2019). These data indicate different roles of *UvCBP1* paralogs during *U. virens* infection of rice flowers, although they may possess similar biochemical functions as suggested by phylogenetic analysis (Figure 8A). In future work, it would be interesting to investigate how *UvCBP1* and its paralogs coordinately support successful colonization and false smut ball formation of *U. virens*.

## Data Availability Statement

The original contributions presented in the study are included in the article/Supplementary Material, further inquiries can be directed to the corresponding authors.

## Author Contributions

JF and W-MW conceived and the project. YL and FH contributed to the planning of research. G-BL, JF, J-LW, J-XH, JL, SS, ZG, X-HH, YZ, and HW performed the experiments and analyzed the data. S-XZ, Y-PJ, MP, J-HZ, Z-XZ, J-WZ, and Y-YH analyzed the data. JF, G-BL, and W-MW wrote the manuscript with input from other authors. All authors read and approved the final manuscript.

## Conflict of Interest

The authors declare that the research was conducted in the absence of any commercial or financial relationships that could be construed as a potential conflict of interest.

## Publisher’s Note

All claims expressed in this article are solely those of the authors and do not necessarily represent those of their affiliated organizations, or those of the publisher, the editors and the reviewers. Any product that may be evaluated in this article, or claim that may be made by its manufacturer, is not guaranteed or endorsed by the publisher.

## Footnotes

1
https://www.uniprot.org

2
https://itol.embl.de/index.shtml

## Supplementary Material

Supplementary Figure 1 Multiple sequence alignment. Amino acid sequences of UvCBP1, together with MoChia1 (G4MXR2), MpChi (A0A385MIS5), and MrChi (A0A0W0FPC8) retrieved from UniProt database, were aligned by MEGA5.1 with default parameters. Red asterisks indicate chitin-binding residues as reported in MpChi (Fiorin et al., 2018). Red rectangle indicates the signal peptide of UvCBP1. The regions with homology over 50% were boxed, and identical residues were shaded in black.

Supplementary Figure 2 Western blot with anti-GFP antibody for confirmation of protein expression in *N. benthamiana* **(A)** and *Arabidopsis* **(B)**.

Supplementary Figure 3 *In planta* expression of *UvCBP1* suppresses chitin-induced expression of defense-related genes in rice spikelets. Expression of *UvCBP1* **(A)** and defense-related genes **(B)** were examined by RT-qPCR using *OsUbi* as the reference gene. Data are represented as means ± SD of three biological replicates. Asterisk indicates significant difference determined by Student’s *t* test (^∗∗^*P* < 0.01).

Supplementary Table 1 Primers used in this study.
