# Supplementary figures and images for: The Flower-Infecting Fungus Ustilaginoidea virens Subverts Plant Immunity by Secreting a Chitin-Binding Protein
Source: Front Plant Sci. 2021 Aug 6;12:733245. doi: 10.3389/fpls.2021.733245 (PMC8377610; doi:10.3389/fpls.2021.733245)

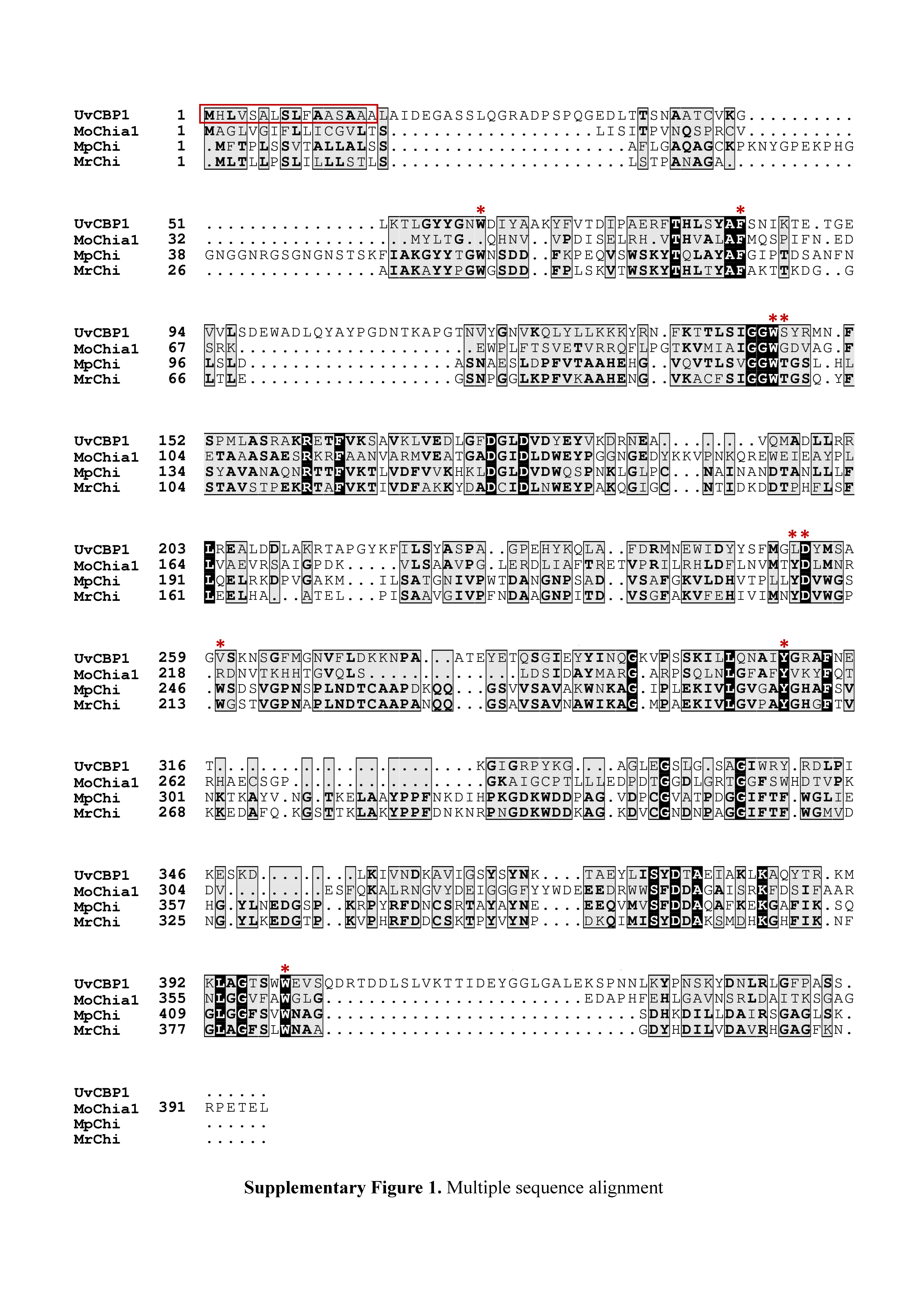

Supplement: Supplementary Figure 1 — Multiple sequence alignment. Amino acid sequences of UvCBP1, together with MoChia1 (G4MXR2), MpChi (A0A385MIS5), and MrChi (A0A0W0FPC8) retrieved from UniProt database, were aligned by MEGA5.1 with default parameters. Red asterisks indicate chitin-binding residues as reported in MpChi (Fiorin et al., 2018). Red rectangle indicates the signal peptide of UvCBP1. The regions with homology over 50% were boxed, and identical residues were shaded in black. [file Image_1.JPEG]

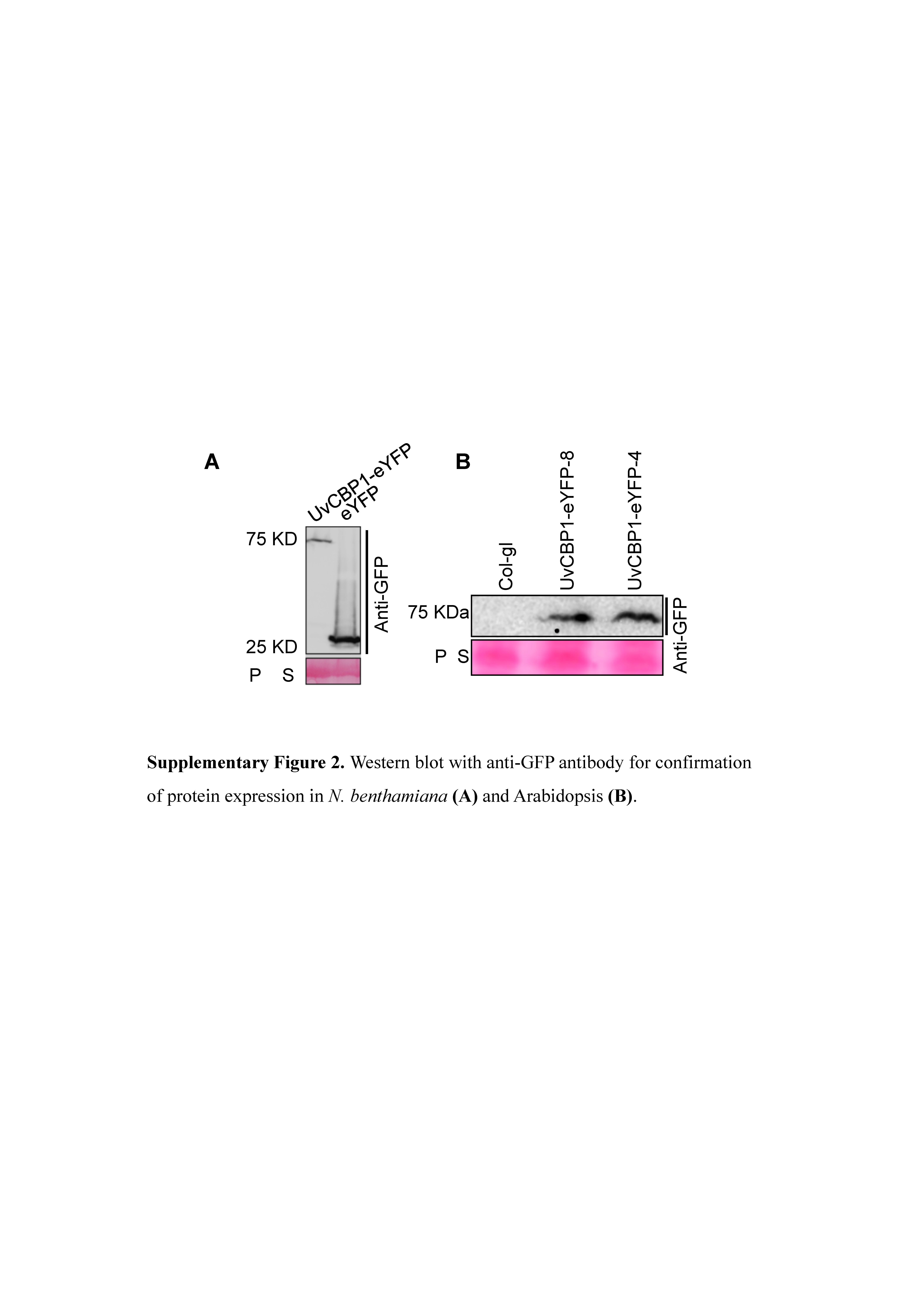

Supplement: Supplementary Figure 2 — Western blot with anti-GFP antibody for confirmation of protein expression in N. benthamiana (A) and Arabidopsis (B). [file Image_2.JPEG]

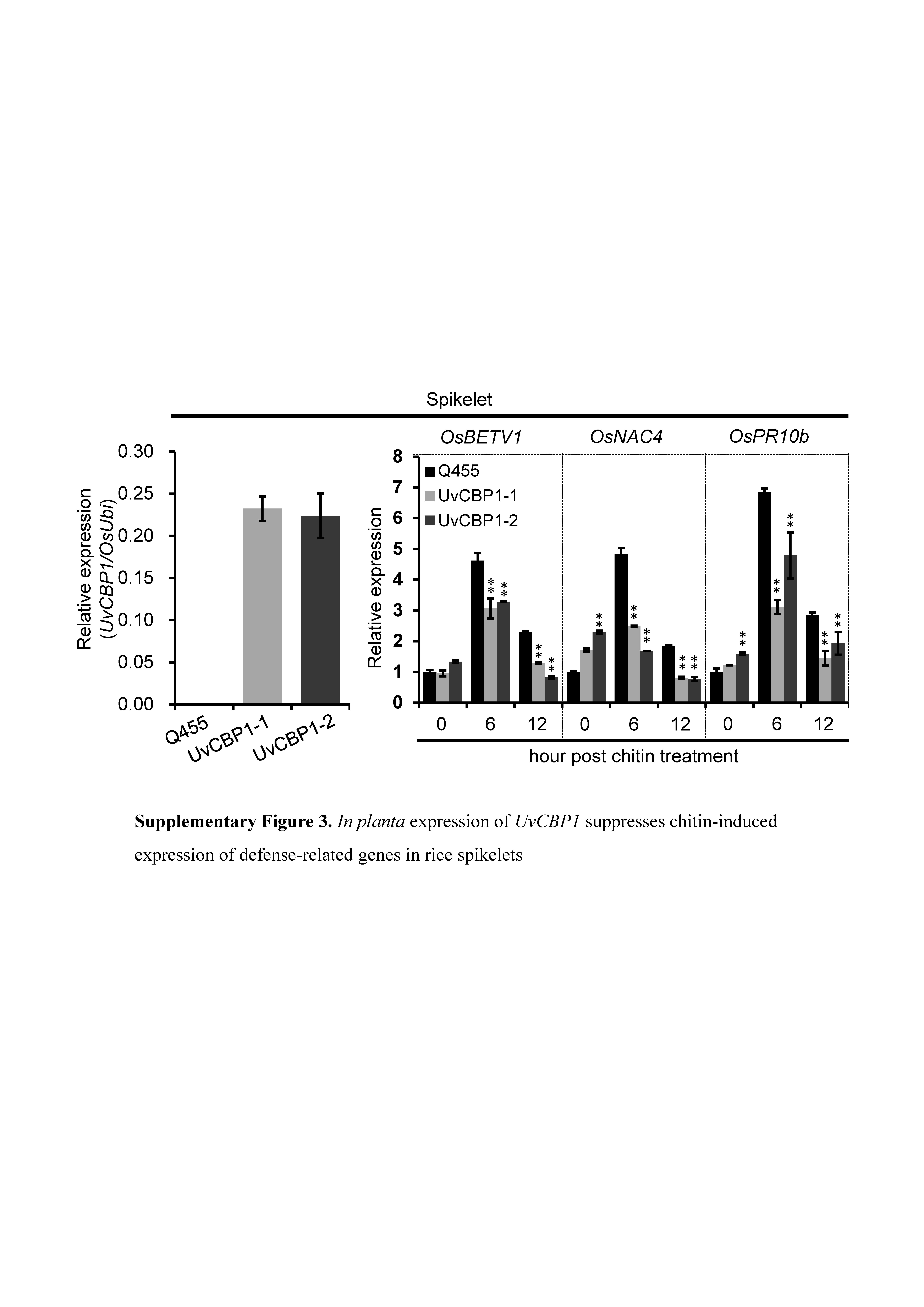

Supplement: Supplementary Figure 3 — In planta expression of UvCBP1 suppresses chitin-induced expression of defense-related genes in rice spikelets. Expression of UvCBP1 (A) and defense-related genes (B) were examined by RT-qPCR using OsUbi as the reference gene. Data are represented as means ± SD of three biological replicates. Asterisk indicates significant difference determined by Student’s t test (∗∗P < 0.01). [file Image_3.JPEG]
